# The Perception of Nurses about Migrants after the COVID-19 Pandemic: Close Contact Improves the Relationship

**DOI:** 10.3390/ijerph20021200

**Published:** 2023-01-10

**Authors:** Paula Berenguel Chacón, Fernando Jesús Plaza del Pino, Brigida Molina-Gallego, María Idoia Ugarte-Gurrutxaga

**Affiliations:** 1 Centre for Migration Studies and Intercultural Relations, University of Almería, 04120 Almería, Spain; 2 Department of Nursing, Physiotherapy and Medicine, University of Almería, 04120 Almería, Spain; 3 Research Group Nursing, Pain and Care (ENDOCU), Campus Tecnológico Fábrica de Armas, University of Castilla la Mancha, 45071 Toledo, Spain; 4 Faculty of Physiotherapy and Nursing, Toledo Campus, University of Castilla la Mancha, 45071 Toledo, Spain

**Keywords:** COVID-19, communication, cultural competence, migrants, nursing, qualitative research

## Abstract

Nurses have faced during the COVID-19 pandemic a tough professional situation in which they have had to work in a resource-limited context and with a high probability of COVID-19 transmission. In today’s multicultural societies, care for immigrant patients is also included. In our study, we have delved into the perception of hospital nurses towards migrant people in the context of the COVID-19 pandemic. We used a qualitative methodology with a phenomenological approach. Semi-structured interviews were conducted with 16 nurses. Seven categories emerged and were distributed in the three blocks of the interviews: (a) perception before the pandemic: prejudices make a mark and communication problems; (b) perception after the pandemic: prejudices weaken and communication worsens; and (c) how to improve care: improve communication, more nurses, and no need for training. The approach in the interpersonal relationship between nurses and patients during the pandemic has improved health care. Proposals arise to overcome the language barrier such as the incorporation of intercultural translators-mediators and professionals of foreign origin. There is a lack of awareness of the need for training in cultural competence on the part of the nurses in the study.

## 1. Introduction

The increase in immigration in Spain has equalized its degree of multiculturalism with countries that receive more immigrants. According to the data from the Instituto Nacional de Estadistica (National Statistics Institute) [1], as of 1 January 2021, a total of 8,472,407 individuals were registered in Andalusia, of which 711,916 were foreign-born. However, the data consulted in the year 2019 showed a total registration of 655,555 foreign individuals. According to the Observatorio Permanente Andaluz de las Migraciones (Permanent Migration Observatory of Andalusia) [2], the distribution of foreigners in Andalusia is unequal. Within the context of our study, we find a greater volume of foreign-born individuals in the province of Almeria after Malaga, out of the total found in the Andalusian autonomous community (19.66%). According to the data from the Instituto Nacional de Estadistica (National Statistics Institute) [3], there is a total of 154,595 foreign-born individuals living in the province of Almeria.

The Observatorio Permanente Andaluz de las Migraciones [2] indicates that the native Andalusian population accepts migrants, with the condition that they adopt the lifestyles of the country. Nevertheless, the studies by Rubio Velasco [4] sustain that the view of society on immigration is negative, due to the reasoning that immigrants make use of the health care system excessively. The arrival of immigrants is usually seen by the host country as a problem, instead of a solution for the social and economic needs of the people who emigrate and of both societies. This perception could result in fear and even rejection, as well as negative preconceptions. On many occasions, the immigrants could suffer marginalization and exclusion in our society, which could lead to situations of conflict, tension, discriminatory behaviors, and self-imposed marginalization, hindering intercultural adaptation, co-existence, and dialogue [5,6].

The representation of migrants as carriers of diseases is one of the most common stereotypes in relation to the migration phenomenon. There are many clear examples throughout history, such as cholera being called the “Irish disease” and the appearance of HIV/AIDS being associated to immigration from Haiti in the USA. In addition, during the COVID-19 pandemic, many examples were observed of the association between migration and the virus in many countries, and Spain was not an exception [7].

During the COVID-19 pandemic, the flow of immigration drastically decreased for the first time in many years, ending the hopes of millions of possible migrants [5]. Additionally, the pandemic had a strong repercussion on those who had already migrated. COVID-19 had a very strong effect on many sectors, especially health care and the economy, as the migrant population is part of the groups defined as fragile, mainly due to their living conditions, and because of being in the front lines of the management of the disease [8]. Among the main hoaxes that appeared in the general population with respect to immigration, due to the reigning disinformation, was the belief that migrants were the main focus of COVID-19 infection [7], aggravating the situation of discrimination already suffered by them [9].

The health and social situation provoked by COVID-19, also known as SARS-CoV-2, slowed the world’s economy and led to the closure of international borders [10]. Spain imposed a state of alarm, resorting to confinement at home, except for special cases such as the so-called essential professions, to stop the propagation of infection [11]. This confinement underlined the essential work performed by migrants, who were now considered indispensable [12].

According to Bellver Capella [13], the pandemic increased the ethical dilemmas that nurses had to deal with such as providing adequate care to the patient, thereby protecting their rights, in a context of limited resources, and with a high probability of COVID-19 transmission. Savitsky et al. [14] argued that nursing professionals were in the frontlines of the battle, despite the lack of material and human resources, together with an emotional overload. Despite the high-risk situation, nurses did not set aside their ethical values, but instead upheld them. However, we have not found studies that focused on the migrant population in the context of a pandemic.

Given the above, the objective of the present study was to analyze the perception of nursing professionals towards migrants, more specifically, within the context of the COVID-19 pandemic.

## 2. Materials and Methods

### 2.1. The Design

We used a qualitative methodology with a phenomenological approach, as it is the most adequate method for delving into the level of discourses and to achieve a deep and rich understanding of experiences from the perspectives of those who endured them [15]. In our case, this method was used to explore the perceptions of nurses on their migrant patients after the context of the COVID-19 pandemic. For this, the Consolidated Criteria for Reporting Qualitative Research (COREQ) were followed [16].

### 2.2. Participants

A purposive sampling method was utilized based on our object of study. The researchers selected key informants, and at the same time identified other participants following the snowball method. The inclusion criteria were Almeria hospital nurses who had had migrant patients before and during the COVID-19 pandemic, and who were still working in the sector during the study. The exclusion criteria were nurses who did not provide an informed consent form, who did not work in Almeria, who did not have migrant patients before and/or during the pandemic, or who were retired or unemployed. Twenty-one nurses were invited to participate in the study, five of whom refused due to time constraints. Interviews were given until the research team decided that data saturation had been reached [17], after which the data collection ended.

Ultimately, a total of sixteen nurses were selected, who worked in different hospital departments in Almeria, such as emergencies, surgery, neurology, ICU, internal medicine, or mental health.

The distribution of the informants, according to sex, age, years of professional experience, and area of service, is shown in Table 1.

The sample shows the feminization of the nursing profession, with only four men participating in our study. The mean age was 35 years old (SD = 5.75), and the mean number of years of professional experience was 12.25 years (SD = 5.65).

### 2.3. Data Collection

The data were collected between January and March 2022, through semi-structured interviews focused on our object of study. The interview was organized into three blocks: (a) perception of the migrant patients before the pandemic, (b) perception of the migrants after the pandemic, and (c) proposal of improvement in the care of migrant patients at the hospital. The interviews were conducted at the place chosen by each participant; in some cases, in a room set up at the hospital for this, and in others, at the nurse’s home. The interviews lasted 35 min on average. All the interviews were recorded with the verbal consent of each participant.

### 2.4. Data Analysis

The interviews were transcribed and reviewed by two researchers. A thematic analysis was performed of the qualitative data. The data were stored, managed, classified, and organized with the qualitative data analysis software ATLAS.ti 22 (https://atlasti.com/updates). An iterative reading of the transcriptions was conducted, after which the codes of meaning were identified, which were later grouped into categories and sub-categories with which to work [18]. The categories identified were aligned to the different blocks proposed in the interview.

### 2.5. Ethical Considerations

The study followed the guidelines established in the Declaration of Helsinki. The approval from the Ethics Research Committee from the Department of Nursing, Physical Therapy, and Medicine was obtained (ENF 162/2021). The data were organized so that the identity, integrity, and access to the participant’s files were protected. The informed consent was solicited from all the participants. The participants were provided with information relevant to the study before the informed consent form was signed. The rights to privacy of the participants were respected, in accordance with Organic Law 3/5 December 2018, on the Protection of Personal Data and Guarantee of Digital Rights, and the data were not utilized for other purposes other than that established in the study objectives.

## 3. Results

After the analysis of the data, about thirty codes were obtained, which were grouped into seven categories distributed in the three interview blocks. Table 2 shows the system of categories, sub-categories, and codes.

The main categories that emerged from the open-ended questions, and their corresponding categories, supported by the narratives of the participants, are described below.

### 3.1. Perception of the Migrant Patients before the Pandemic

In this section, we present all the comments of our informants about their migrant patients, focusing on their experiences before the start of the COVID-19 pandemic. The relationship and view of these patients were conditioned by the prejudices they have, and the existence of a language barrier.

#### 3.1.1. The Prejudices Make a Mark

The nurses’ comments denoted the existence of social prejudices and stereotypes that determined the interpretation of culture, their expressions, and behaviors.

- View of cultural aspects.

The interpretation of culture is established as a function of the gender stereotypes or assumptions that try to explain the other’s behavior, in many cases, differentiating the way to act according to the patient’s origin.

*The women were very submissive with the men, due to their culture, the one who is in charge is he, the one who decides everything*.

(Nur4)

*On many occasions, I’ve been taking a blood sample, and especially the “negritos” (little black ones), they don’t want it because for them, drawing blood out is bad*.

(Nur6)

*They see the English as people who come here to have fun, the Arabians as those who are here to steal our jobs, and those that come from the center of Africa, the darker ones, they think of them as “how sad”*.

(Nur5)

- Forms of expression.

Discriminatory expressions were recorded throughout the interviews, such as the use of diminutives (as in the verbatim phrase above: negritos) or derogatory terms such as “moro”, together with pejorative comments.

*The Moors have been here for a long time and don’t speak Spanish…they know the laws better than you and me*.

(Nur13)

- Poor treatment.

With respect to the assessment of their manners towards their migrant patients, they confess that on many occasions, it is discriminatory, excusing these behaviors with the high workloads they are subjected to.

*In general, almost everyone in my floor is a racist, so that if there’s a lot of work and you have to decide, you dedicate less time to the immigrants*.

(Nur12)

Likewise, they state that they believe that there are no discriminatory attitudes, but rather a greater involvement with these patients, although it is more difficult given the high pressure on the health care system.

*I believe that the treatment is good, and sometimes even great. We are even more aware*.

(Nur15)

*At a hospital, it is complicated, there are a lot of people, and in the end, it is difficult to provide good care, you suffer lots of stress, and in the end, it results in dehumanization*.

(Nur12)

- Use of health services.

Some of the nurses commented on the “abuse” of the emergency services and the responsibility of the migrants for the saturation of the health system.

*Before the pandemic, many immigrants came to emergency services, for anything. No health system is able to manage*.

(Nur14)

#### 3.1.2. Communication Problems

In all the interviews, we found the recurring problem of communication related with the language barrier with the migrant patients.

- Consequences of the lack of communication.

The language barrier determines the development of the therapeutic relationship and worsens the quality of health care with possible diagnostic errors and risk for the safety of the patient.

*During the classification consultation (in emergency services), he tells you or points to his back or leg, and then we say “but does it hurt”, and he says yes, you ask the opposite and says the same thing, you realize that they don’t understand anything*.

(Nur1)

*You have to be very careful when you have to give an intravenous drug or a pill, you never know if they are allergic or not*.

(Nur7)

- Nurses’ concerns: seek solutions.

The nurses are aware of the importance of overcoming the language barrier, so they seek alternatives given the lack of formal translators at the hospital. The main tools they use are informal translators (any person that can translate, independently if they are family members of the patient or not), pictograms, or non-verbal communication.

*What we do is to use any companion if they speak Spanish, or any person from the Maghreb available*.

(Nur12)

*We have pictograms with very basic things, it’s very limited*.

(Nur5)

*We usually believe that if they speak another language, they will understand if we speak louder. With gestures, facial expressions, and speaking correctly, we understand each other a bit more*.

(Nur2)

### 3.2. Perception of the Migrant Patients after the Pandemic

The second block addresses experiences with the migrant patients during the pandemic and how they have affected (or not) their perception of these patients. Two categories appeared related to the change in the prejudices and impossible situations of non-communication.

#### 3.2.1. The Prejudices Weaken

The nurses coincided in that their perception of the migrant patients changed after the difficult situations experienced during the pandemic.

- Contact increases: the relationship improves.

Our informants indicated that during the pandemic, there was a greater relationship with the migrant patients, especially in the case of the women. Patients have experienced “a pandemic without family” in the hospital; the fact that they could be accompanied by anyone due to the confinement measures led to a closer relationship.

*The pandemic has made us become closer*.

(Nur2)

*I was able to have a closer relationship, we had more chances to meet, for them to explain things in more detail, now I see them in a different light*.

(Nur10)

*There was a moment in time when you didn’t have any appointments, and I’ve seen colleagues who made an extra effort with them (…), or you go in to speak to them, and try to give encouragement or set up the camera so they can talk with their families…and yes, I think it has united us a lot*.

(Nur7)

The nurses mentioned that given the complicated situation they were sharing, there was a greater interest in becoming closer to the patient despite the language barriers and the limited time they had available, which changed their view of these patients.

*It’s been really hard for everyone, also, they don’t have family here, and they almost don’t understand (…) the pandemic, well, yes, truth is, it changed me when I saw them in that way*.

(Nur11)

*It’s true that you have some prejudices, and when you deal with people from other cultures, your perspective changes*.

(Nur8)

#### 3.2.2. Communication Worsens

The nurses highlighted that the main difficulty they had with the migrant patients was the language barrier, which was aggravated during the pandemic.

- Few solutions for the lack of communication.

Not being able to rely on informal translators due to the isolation measures and the added difficulty in expressing oneself with the personal protection equipment (PPE) and the face masks resulted in the alternatives to the lack of communication becoming limited to tele-translation, which was not always available.

*In the ICU, with the Covid (patients), we had a Moroccan patient who had had an operation, and the surgeons did not know how to explain well what they had done, and of course, the man was desperate, he did not understand anything and no one was able to translate*.

(Nur7)

*The pandemic has caused great havoc when you cannot communicate, not even with gestures, with everything we are wearing, it was either phone translation or nothing. We only used it when we had to say something important, because it was hard to do. There were moments in which you suffered…*.

(Nur2)

Some informants believed that the lack of communication situation experienced during the pandemic could have been solved if the health services had their own translators.

*Everyone knew what was going on and no one did anything. If we had had our own translators, everything would have been different*.

(Nur3)

The individuals who were interviewed believed that the lack of translation aggravated an already complicated situation that health professionals and migrants were experiencing with COVID-19.

*For me, the worst thing was the lack of communication*.

(Nur5)

*Because if you are alone and can’t speak the language, how do you do it? You suffer more with everything*.

(Nur3)

### 3.3. How to Improve the Care for Migrant Patients

For the last part of the interview, the nurses were asked to suggest measures that could be implemented to improve the care given to migrant patients to avoid the complicated situations experienced during the pandemic. The proposals were centered on ending the language barrier and having more human resources available. Only one informant mentioned the need for intercultural training.

- Improve communication.

To overcome the language barrier, the nurses proposed the implementation of the figure of intercultural mediator-translator at the hospital to achieve an efficient translation with the migrant patients. They also explained the benefits of having migrant professionals at the hospital they could rely on when needed.

*I would hire someone, even part-time, who spoke the language, that is, who could serve as a wildcard that we could use for the different consultations…when there is a problem, we could call on him*.

(Nur1)

*It would be very important to have people who master the language. We had an Arabic man who was a (nurse’s) aid, and when he was with us, it was awesome, because the doctors called on him, we called him…*.

(Nur3)

They also mentioned the need to develop their own skills to improve communication with their migrant patients.

*Communication is broken. Perhaps not enough importance is given, but how you say things, the gestures you make, everything is important, and we don’t know how to do it. We could also learn*.

(Enf1)

- More nurses.

Our informants mentioned the lack of nurses and the high workloads as a handicap that impedes them from providing good care to patients who could need more time, such as migrants. The hiring of more nurses would make possible the offering of a comprehensive approach to migrant patients.

*To be able to give more time to the patients. Because between the bad communication and the concerns we always have, it is impossible*.

(Nur14)

*To be able to speak with the families calmly, and follow up the cases once they are released. Then, they have appointments and they don’t come, who knows why*.

(Nur8)

- No need for intercultural training.

It was interesting to see that only one nurse mentioned the need for intercultural training to improve the care of migrant patients, beyond that mentioned by other informants with respect to the improvement in communication skills.

*But yeah, training, the thing is, we can’t always provide standardized care, we have to learn how to do it*.

(Nur4)

## 4. Discussion

The present study explores the perception of nurses about migrants within the context of the COVID-19 pandemic, and how these perceptions could have affected their interactions with this population [19]. Our results highlight two important themes in the nurses’ perception of migrant patients: the importance of prejudice in the relationship and communication problems due to the language barrier. Both themes have been modified after the pandemic situation.

Negative attitudes towards migrant patients are one of the themes that emerged during the analysis in our study. Discrimination in the health system is a worrying matter at the international level, as it has a direct effect on both the access and use of the health care system (e.g., following programs, therapeutic adherence), as well as the health of the migrant patients [20,21,22,23,24,25]. In fact, the development of actions to address racism and racial discrimination in health systems was selected as an important subject in the 76th session of the UN General Assembly in 2021.

In the current social panorama, it is a reality that although the advantages related with migration are evident, such as a potential solution of the scarcity forecasted of working age populations in countries with an aging population [19], in the social plane, and among health professionals, we find discourses full of prejudices and stereotypes directed towards migrants, with presupposed violent and threatening behaviors, carriers of infectious diseases, and guilty of the overload in the social and health system in the host countries [19,26,27].

In our study, we also found the rise of this preconception of abuse of migrants of the health system, making them responsible for the saturation in health centers [28]. This idea is far from the truth, as shown by the studies conducted on these aspects. According to García González [29], these are specific cases of what is called health tourism. In most cases, these are individuals who escape their countries due to war or because of a lack of compliance with human rights. Additionally, according to this study, the immigrant population uses the health system to a lesser degree as compared to the national population. For this, equalizing a health tourist with an irregular immigrant is xenophobic.

Additionally, in this study, we found discriminatory expressions throughout the interviews, and the use of diminutives or derogatory words such as “moor”, together with pejorative comments. It is frequent to find racist comments towards immigrant populations from health professionals, but referring to others, not to themselves [30]. With respect to the use of diminutives in the patient–health professional relationship, although it could be interpreted as a sign of closeness and trust [31], it is still an example of health care paternalism and infantilization of the patient. Many examples of this type of treatment have been found in different articles on migration and sexual and reproductive education, especially when the patients are women and migrants [32].

Along this line, a deficient treatment was recognized in the present study towards the migrant population, although it was justified by the high workload of the health professionals. It is true that the health care encounter took place in a context of a systemic lack of time [33], which made difficult the providing of care, especially considering the communication difficulties due to language and cultural reasons. This situation, which on many occasions came from dehumanization when dealing with migrants, goes against their right of protection of health and access to health services [34]. It has been shown that the nurses’ words and behaviors can involuntarily dehumanize patients [35,36].

Additionally, the migrant population is culturalized. Cultural bias is the exaggeration of the cultural factors and the essentialization of the culture. The consequence of this bias is the interpretation of people’s behaviors due to their membership to a specific culture. As Aguilar and Burashi commented [37], on many occasions, people interpret the behavior of individuals only due to their belonging to a specific culture, “mistaking the social differences with the cultural differences”. An example of this cultural bias is assuming that all women from an Islamic religion are quiet in the consultation due to being female, because it is expected by her religion, when in fact, being silent could be due to language difficulties (social inequality). It is true that in Islamic countries, gender equality is low, but this gender inequality is also found in countries that are predominantly Christian [38].

Another one of the themes that emerged in the discourse from the participants was the concern with the language barrier, which can condition the diagnosis and the therapeutic adherence of the migrant patients. The migrant’s difficulties with the language have been widely documented in the scientific literature [39,40,41]. The nurses who were interviewed, aware of the importance of overcoming the language barrier, utilize informal translators, pictograms, or non-verbal language. However, in our case, and due to the pandemic, in which health care was provided mostly through computers or telephones, nurses seemed to be more aware that to overcome language difficulties, in-person care was fundamental, and they especially requested intercultural translators-mediators. Many studies have verified that the addition of professional interpreters in the cases in which the language barrier impedes meaningful communication between migrants and health professionals provides more safety and fidelity in communication and guarantees the confidentiality to which the patients have a right [42,43].

Despite finding the scientific literature that supports the idea that the main barriers to access is due to the lack of acknowledgement of the migrant population as legal subjects, and at the same time to the nurses not thinking of themselves as guarantors of the rights of this population group they provide care for [44], and despite the scarcity of the studies, it seems that the pandemic has provoked a greater confrontation in their work with the patient [45]. In our case, and due to the COVID-19 pandemic, we saw that nurses changed the type of interpersonal relationships with migrants. In the especially severe health situation provoked by the pandemic, they felt a greater closeness between the health professional and the patient. A similar situation was observed in the study by Sevinç [46] which focused on the experiences of nurses who worked in a Turkish internal medicine clinic with Syrian refugees, in which the nurses found it difficult to communicate with the Syrian refugees and their families at the clinic. They observed and experienced differences and similarities in the care of Turkish and Syrian patients and showed compassion towards the Syrian refugees when providing care.

Compassion in health care, defined as the awareness and openness towards one’s own suffering and that of others, together with the motivation for alleviating and preventing it [47,48], is one of the subjects that is currently garnering great interest [49,50]. Many authors and researchers have suggested that the perception of common humanity is the basis of compassion [47,51]. Starting with this premise, in our case, it seems that the common suffering experienced during the pandemic by both parties (nurses and patients) has resulted in a feeling of affiliation between them [52]. The impossibility, due to the confinement measures, that patients could bring companions (e.g., family, friends) implies a closer and more intimate treatment between nurse and patient, and this closeness allowed them to dismantle many of the prejudices they had about the migrant population until then. Due to this rapprochement, the nurses became aware that communication was deficient through telephone-based translation, and that the lack of translators could result in misunderstandings, leaving them feeling impotent. This feeling is coherent with the idea that communication in health matters, and especially during times of epidemiological crisis, must be direct, and adapted to each socio-cultural context of the population, with non-verbal communication playing a fundamental role [53].

Due to this situation, which provoked so much discomfort, the nurses proposed measures that could favor communication. Among them, we found the incorporation of formal and professional migrant interpreters-translators. The incorporation of the intercultural mediator figure, as compared to the use of other individuals (e.g., family, friends) who facilitate translation, was one of the proposals provided to overcome the language barrier. We found numerous studies that provided the same results in this sense [54,55,56,57].

Another one of the proposals suggested to improve communication during the health care-related visit was the addition of migrant professionals. This proposal is not confined to our study, but it is also found in other studies [58], with special emphasis on the hiring of international nursing personnel [59,60]. These studies argue that the incorporation of nurses from other cultures is a key aspect for providing culturally competent care to patients and populations that are ever more diverse, and this strategy could improve the access to health care services and decrease health inequalities [61,62].

To conclude, we must mention that surprisingly only one of the nurses interviewed manifested her interest on intercultural training in nursing. We agree with the statement by Larenas-Rosa citing Pedrero et al. when the author says that “many of the health professionals do not have cultural awareness that makes the teams aware of their cultural values and constructions; they do not value the culture of the other, or take action from one’s own culture; they do not value the experience of the other, nor recognize the other’s social context, or life process, aspects that as a whole could improve the understanding of the complexity of the other and the interaction” (136, [63]).

We found abundant scientific literature that considered cultural competency as a key strategy in the care of migrant populations that are culturally diverse [64,65,66,67]. Evidence was found that training on cultural competence increases knowledge about other cultural groups [68,69], and increases cultural awareness and skills [70], all of which contributes to the decrease in confrontations between health professionals and patients and the improvement in health care.

In fact, there are many training proposal experiences for nurses centered on cultural competence in health care [71,72,73].

## 5. Conclusions

Racism by health professionals, and the population in general, has a long history. This subject is not openly discussed in this collective, perhaps due to a social desirability effect. A strong recommendation is made to conduct studies to identify the underlying causes of racism in the health care context. Discriminatory treatment can be considered as dehumanizing treatment, which goes against the fundamental principles of the ethics of nursing care. Therefore, more studies should be conducted to explore the association between dehumanizing behaviors of the nurses and their impact on care provision.

Another one of the emerging themes was the concern about the language barrier when providing care. At first, the nurses tried to overcome this barrier through the use of telephone-based translators, pictograms, gesture-based communication, and informal translators, who are on many occasions family members of the patients. However, there is a very significant difference in this approach during and after the COVID-19 pandemic.

On the other hand, the closer interpersonal relationships between nurses and patients, as a result of the pandemic, is a hopeful sign for the improvement of health care. The awareness and openness toward common suffering implies a significant change in nurses, from which new proposals emerged to overcome the language barrier, as in the case for their interest in the incorporation of formal translators-mediators. This measure implies a structural adaptation of the health system, which could result in greater quality care. Another of the proposals that emerged was the addition of foreign-born professionals. This practice has been little explored in Spain, and we believe it is now necessary to do so.

To conclude, we must state that we found a lack of awareness of the need for cultural competence training by the nurses in our study. We believe that the development of cultural competence can facilitate the awareness of the professionals about their behaviors, the biases (cultural), and the discrimination in health care, and also contribute to the shaping of meaningful health care for migrants.

## Figures and Tables

**Table 1:** Data of the informants.

CODE	SEX	AGE	YEARS OF EXPERIENCE	HOSPITAL SERVICE
Nur1	Female	40	18	Emergencies
Nur2	Female	32	10	Surgery
Nur3	Male	44	21	Emergencies
Nur4	Female	42	14	Surgery
Nur5	Female	26	3	Neurosurgery
Nur6	Female	50	29	Digestion
Nur7	Female	40	19	ICU
Nur8	Female	38	16	Mental Health
Nur9	Male	31	9	Internal Medicine
Nur10	Female	29	7	Gynecology
Nur11	Male	28	6	ICU
Nur12	Female	37	7	Internal Medicine
Nur13	Female	27	5	Traumatology
Nur14	Male	30	8	Emergencies
Nur15	Female	36	14	Internal Medicine
Nur16	Female	32	10	ICU

**Table 2:** Comprehensive list of categories identified after content analysis.

Categories	Sub-Categories	Codes
Perception of the migrant patients before the pandemic		
The prejudices make a mark	View of cultural aspects	Way to act. Gender relations.
	Forms of expression	Giving names to the migrant: moor, “negrito”. Xenophobic comments.
	Poor treatment	Excuse: high workloads
	Use of health services	Abuse. Responsible for saturation.
Communication problems	Consequences of the lack of communication	Poor health care: diagnostic errors, lack of patient safety.
	Nurses’ concerns: seek solutions	Informal translators. Non-verbal communication. Tele-translation. Pictograms.
Perception of the migrant patients after the pandemic		
The prejudices weaken	Contact increases: the relationship improves	Share the pandemic without a family. More interpersonal relations. More empathy.
Communication worsens	Few solutions for the lack of communication	Without informal translators. Non-verbal communication with face masks and PPEs. Only tele-translation. What if we had formal translators?
How to improve the care for migrant patients		
	Improve communication	Intercultural/professional mediators of migrant origin. Communication skills.
	More nurses	To have more time. To be able to provide a comprehensive approach.
	No need for intercultural training	

## Data Availability

The data presented in this study are available upon request from the corresponding author.
